# Informative data visualization with raincloud plots in JASP

**DOI:** 10.3758/s13428-025-02773-5

**Published:** 2025-08-18

**Authors:** Vincent L. Ott, Don van den Bergh, Bruno Boutin, Johnny van Doorn, František Bartoš, Nicholas Judd, Jordy van Langen, Luke Korthals, Rogier Kievit, Laura Groot, Eric-Jan Wagenmakers

**Affiliations:** 1 Department of Psychological Methods, University of Amsterdam, Nieuwe Achtergracht 129B, 1001 NK Amsterdam, The Netherlands; 2 Donders Institute, Radboud University, Nijmegen, Netherlands

**Keywords:** Anscombe’s quartet, Good research practices, Statistical software

## Abstract

Proper data visualization helps researchers draw correct conclusions from their data and facilitates a more complete and transparent report of the results. In factorial designs, so-called *raincloud plots* have recently attracted attention as a particularly informative data visualization technique; raincloud plots can simultaneously show summary statistics (i.e., a box plot), a density estimate (i.e., the cloud), and the individual data points (i.e., the raindrops). Here we first present a ‘raincloud quartet’ that underscores the added value of raincloud plots over the traditional presentation of means and confidence intervals. The added value of raincloud plots appears to be increasingly recognized: a focused literature review of plots in *Psychonomic Bulletin & Review* shows that 9% of plots in 2023 were raincloud plots. Another 29% of plots (vs. 2% in 2013) contained individual data points (i.e., raindrops), indicating a strong trend towards transparent and informative data visualization. To further encourage this trend and make raincloud plotting easy and practical for a broader group of researchers and students, we implemented a comprehensive suite of raincloud plots in JASP, an open-source statistics program with an intuitive graphical user interface. Examples from two factorial research designs illustrate how the JASP raincloud plots support a correct and comprehensive interpretation of the data.

Graphical output [...] is readily available to anyone who does [their] own programming. [...] Unfortunately, most persons who have recourse to a computer for statistical analysis of data are not much interested either in computer programming or in statistical method, being primarily concerned with their own proper business. Hence, the common use of library programs and various statistical packages. Most of these originated in the pre-visual era. The user is not showered with graphical displays. He can get them only with trouble, cunning, and a fighting spirit. It’s time that was changed.

Anscombe, 1973, p. 21

Data visualization is crucial for a correct and complete understanding of both experimental and observational data (e.g., Tukey, 1972, 1977). Students who take courses in data analysis are traditionally confronted mostly with the fundamentals of statistical modeling, and this may create the temptation to apply the statistical models thoughtlessly, under the untested assumption that summary statistics paint a complete picture of the underlying data. The pitfalls of relying exclusively on summary statistics are aptly illustrated by ‘Anscombe’s quartet’ (Anscombe, 1973) shown in Fig. 1. The quartet consists of four scatter plots, each comprised of 11 pairs of observations. The observations have been constructed such that key summary statistics are identical across the four plots; specifically, in each scatter plot the two variables *x* and *y* have the same sample mean (i.e., $\text {M}_x = 9.00$ and $\text {M}_y = 7.50$, respectively) and the same sample standard deviation (i.e., $\text {SD}_x = 3.32$ and $\text {SD}_y = 2.03$, respectively). Moreover, the Pearson sample product-moment correlation coefficient equals $r_{xy}=.816$ in all four panels, suggesting a strong positive relationship between the two variables (cf. Fig. 1 in van Doorn et al. , 2021). However, a mere glance at the four scatter plots reveals that the inference is valid only for the scatter plot from the top-left panel, where the observations appear randomly dispersed around the linear regression line.

**Fig. 1:**
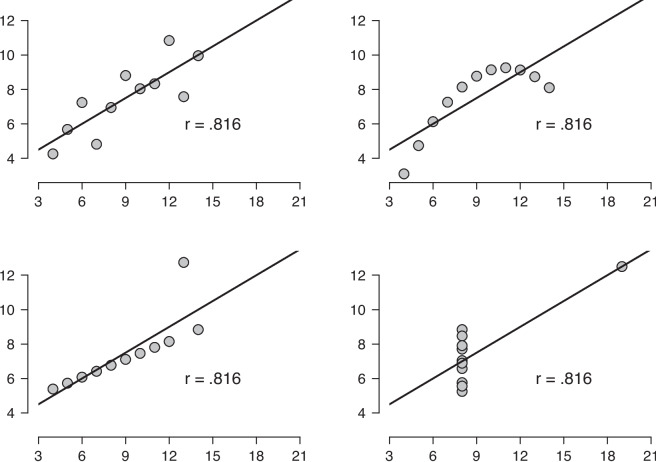
Anscombe’s quartet highlights the importance of data visualization for correlations. Conclusions based on the Pearson product–moment correlation are meaningful only for the *top-left* scatter plot. Figure created using R code from Wagenmakers and Gronau (n.d.)

In the top-right panel, the relation is quadratic; in the bottom-left panel, an otherwise perfect linear relation is perturbed by a single outlier; and in the bottom-right panel, another outlier drives the entire effect. Anscombe’s quartet shows that it is hazardous – perhaps even reckless – to draw conclusions about correlations without visually examining the scatter plot first.

In general, data visualization allows researchers to confirm that their conclusions do not hinge on a seriously misspecified statistical model. Data visualization is also an essential element of a comprehensive and transparent research report, simultaneously generating trust and inviting appropriate scrutiny from the academic community (Riedel et al., 2022; Weissgerber et al., 2015, 2019; Loffing, 2022; Drummond & Vowler, 2011; Nimphius & Jordan, 2020; Teare, 2016; Psychonomic Society, 2019).

**Fig. 2:**
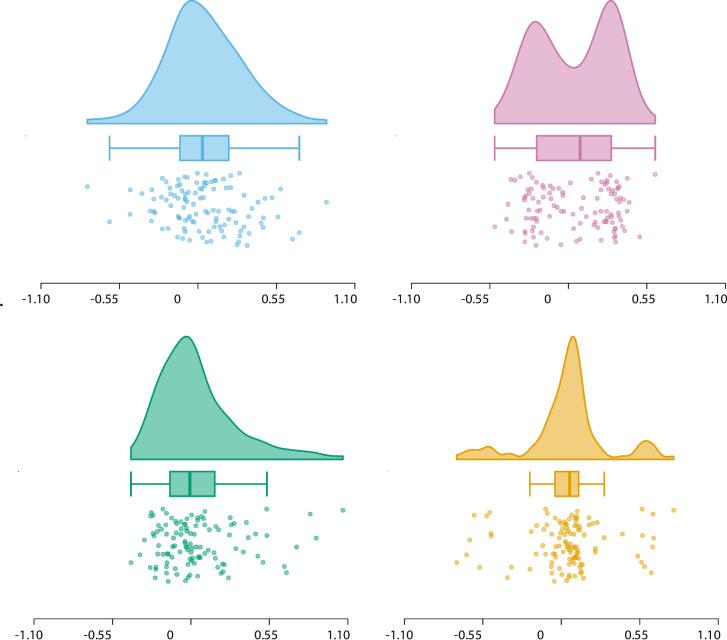
A raincloud version of Anscombe’s quartet highlights the importance of data visualization for factorial designs. Figure from JASP (JASP Team, 2024), colors from Okabe and Ito (2008)

In experimental work, the primary concern often lies not so much with correlations between two continuous variables but with possible changes in a dependent variable across conditions or groups. For such factorial research designs, a data visualization method that has recently attracted attention is the raincloud plot (Allen et al., 2021). The essential element of the raincloud plot is that it shows the individual observations (i.e., as raindrops). At the same time, the raincloud plot also shows a density estimate of the entire distribution (i.e., as the cloud itself), and summary statistical information as conveyed by a box-and-whiskers plot. Together, raincloud plots provide a complete overview of the data and accommodate “inference at a glance”.

To underscore the importance of data visualization for factorial designs and highlight the conceptual similarity with Anscombe’s demonstration for correlations, Fig. 2 presents a raincloud quartet^1^ (cf. Fig. 1 in Stuart et al. , 2024). Crucially, the data in each of the four panels of the raincloud quartet were created such that popular sample statistics are identical. Specifically, in each of the four panels, the sample mean equals $\text {M} = 0.04$ and the sample standard deviation equals $\text {SD} = 0.27$; together with a sample size of $\text {N}=111$ this implies that in each panel, a one-sided one-sample *t*-test yields the exact same inference; a frequentist analysis shows the result to be statistically significant (i.e., $t(110) = 1.67$, $p =.049$; 95% CI for Cohen’s $\delta$ = [-0.03, 0.35]), whereas a default Bayesian analysis yields $\text {BF}_{0+} \approx 1.31$, indicating that the data are slightly more likely under the null hypothesis than under the alternative hypothesis in which effect size $\delta$ is assigned a positive-only Cauchy distribution with scale parameter of 0.707 (cf. Jeffreys , 1961; Ly et al. , 2016; Wagenmakers and Ly , 2023).

However, as is the case for Anscombe’s quartet, the inference is only meaningful for the data from the top-left panel, where the data are approximately normally distributed. In contrast, the data from the top-right panel are bimodal, the data from the bottom-left panel are right-skewed, and the data from the bottom-right panel are riddled with outliers. The raincloud plots reveal these qualitative differences immediately, preventing researchers from drawing conclusions that are evidently inappropriate, as these depend on a seriously misspecified statistical model. Note that raincloud plots also have advantages over alternative visualizations such as histograms and Q-Q plots. Histograms lack information about summary statistics (e.g., the median), do not show individual observations, and add visual clutter through their bars (unlike a density estimate). Q-Q plots require considerable experience to interpret correctly and hinge on a comparison to a theoretical distribution (usually a normal distribution) – Q-Q plots are not designed to facilitate a visually intuitive assessment of the distribution on its own.

## Raincloud plots emerge: A focused literature review

While raincloud plots facilitate a comprehensive and transparent analysis of data from factorial designs, they have only been introduced relatively recently (i.e., in preprint form: Allen et al.  2018; though see Fig. 2.4 in Ellison , 1993, for a raincloud-similar plot) Therefore, raincloud plots do not yet feature in reviews concerning the types of plots used in the empirical sciences (e.g., Riedel et al.  2022; Weissgerber et al.  2015, 2019). In order to obtain a first indication of the extent to which raincloud plots are gaining traction in experimental cognitive psychology we conducted a focused literature review.

### Method

We reviewed articles published in 2013 and 2023: 5 years before and 5 years after the moment that raincloud plots were introduced in the literature (in preprint form; Allen et al.  2018). Specifically, we considered all Brief Reports published in the 2013 and 2023 volumes of *Psychonomic Bulletin & Review*, the flagship journal of the Psychonomic Society.^2^ In the initial stage, the first author rated for each Brief Report ($\text {N}_{2013} = 121$ and $\text {N}_{2023} = 133$) whether or not the associated data could have been presented as a raincloud plot. Generally, this is possible if the data are continuous and originate from a factorial design. In 2013 and 2023, 82% (i.e., 99/121) and 79% (i.e., 105/133) of the Brief Reports contained data suitable for raincloud plotting, respectively. From this suitable set of articles, actual data were plotted in 82% (81/99) of the cases in 2013, and 90% (94/105) in 2023. These then form the relevant subsets of Brief Reports (i.e., a plot is presented, and it could have been a raincloud plot) for which the first author evaluated the different plot types. When an article contained multiple plots, the plot was evaluated that most closely resembled a raincloud plot. Thus, a single plot was evaluated for each Brief Report.

### Results and discussion

**Table 1:** Raincloud plots emerge. Plot Types from ${\textit{N}}_{2013} = 81$ and ${\textit{N}}_{2023} = 94$ *Psychonomic Bulletin & Review* Brief Reports with Raincloud-plottable Data

	Plot Type					
Year	Bar	Box	Point	Line	Raw data	Raincloud
2013	53% (43)	1% (1)	5% (4)	38% (31)	2% (2)	—
2023	48% (45)	1% (1)	3% (3)	11% (10)	29% (27)	9% (8)

*Note*. Percentages for 2013 (2023) sum to 99% (101%) due to rounding. Point = plotted means as points (i.e., bar plot without bar). Raw data = presents individual observations (i.e., the raindrops) but does not comprise all three elements of a raincloud plot

The results of the focused literature review are presented in Table 1. In both 2013 and 2023, bar plots are relatively popular and occur in about half of the relevant cases, echoing results from other academic disciplines (Riedel et al. , 2022; Weissgerber et al. , 2015, 2019). Of interest here is the prevalence of raincloud plots. In 2013, not a single Brief Report presented a raincloud plot – this is not surprising, since raincloud plots were introduced only in 2018. However, in 2013, we nonetheless identified two Brief Reports that presented plots with raw data, which showed the individual data points (i.e., the raindrops) as well as group-level information. Such plots present an intermediate step towards informative data visualization with raincloud plots. In general, it is evident that in 2013, plotting practice for factorial designs reported in *Psychonomic Bulletin & Review* was dominated by bar plots and line plots. In 2023, this plotting practice has undergone a dramatic transformation: 9% (8/94) of relevant Brief Reports present raincloud plots and another 29% (27/94) display raw data, that is, the individual observations. Examples of the latter include a bar plot with raw data points or a point plot with lines that connect individual observations across conditions.

The steep increase of plots with raw data and the emerging raincloud plots are presumably due to an increasing awareness that data should be visualized transparently (e.g., Riedel et al. , 2022; Psychonomic Society , 2019). In line with this development, raincloud plots are also beginning to appear in popular statistics course books (e.g., Field et al. , 2025). As outlined in the following, we believe that the next step of this development in the empirical sciences is the widespread adoption of raincloud plots through accessible software – especially since the majority of plots are still uninformative bar plots.

## Raincloud plots in JASP: Easy and informative

Raincloud plots visualize data in a transparent and informative way and thus constitute a building block of good research practices. To facilitate their widespread use, raincloud plots should be easy to create *and* have ample functionality. However, up until recently, this was not the case with software for raincloud plotting. Given considerable programming expertise and effort, users could create the plots themselves – but this was not easy (Min and Zhou , 2021; Allen et al. , 2021; Judd et al. , 2024; Poggiali et al. , 2024; for paid software to create raw data plots see Loffing , 2022). These programming demands therefore create a hurdle for researchers and students who lack either programming knowledge or time.

These hurdles can be overcome through a software program equipped with a graphical user interface (GUI). The GUI presents a convenient and efficient way to plot without programming. For example, a GUI allows teachers to include raincloud plots in their teaching even when students have different levels of programming expertise; teachers can focus on the plots without having to explain the programming commands. Moreover, even researchers with considerable programming expertise may find that working with a GUI greatly enhances their efficiency (i.e., the GUI allows an aesthetically pleasing raincloud plot to be produced with a handful of mouse clicks, without having to track down old code and debug new code). Finally, the GUI provides a concrete, explicit series of alternative plotting options that can inspire researchers and students to make their plots more informative.

While some GUI-based web applications have been created to produce raincloud plots (i.e., SuperPlotsOfData^3^ by Goedhart, 2021; raincloudplots^4^ & Raincloud-shiny^5^ by Allen et al., 2021), they lack the functionality to create raincloud plots for a wide range of different scenarios. For example, they do not support flexible data exploration (e.g., visual outlier detection through boxplot whiskers or covariate information) nor flexible data publication (e.g., adding inferential credible/confidence intervals or fine-tuning for more complex designs). In addition, a raincloud GUI should ideally be embedded in a software environment that also supports other statistical activities.

In order to facilitate the broader adoption of raincloud plots, we have now made them easy to produce via the GUI in JASP (JASP Team, 2024). JASP is a free and open-source program for statistical analyses that is used worldwide in research, teaching, and industry.^6^ JASP facilitates reproducible, open science where analysis settings and results are coherently connected and files can be easily annotated and shared (Love et al., 2019). While limited raincloud plotting had been possible in JASP for some time (Lüken, 2021), JASP now has a designated, comprehensive module to create raincloud plots for a wide range of scenarios. For instance, the JASP raincloud plots can visualize up to two factors and one additional covariate that may be continuous or discrete. Means and different uncertainty intervals can be added to the plots. In repeated measures designs, individual trajectories can be shown. Moreover, virtually all plot elements can be fine-tuned for a finishing touch (e.g., element size, distance between elements, or axes). Finally, a table provides key statistics such as sample sizes and group medians. The JASP raincloud plots come with nine color palettes and allow the specification of custom colors. As shown by the present plots, these coloring options not only ensure accessibility for colorblind people but they also aid comprehension since plots that belong together can be grouped by color.

In the following, we present several examples for two popular factorial designs in the empirical sciences that illustrate how the JASP raincloud plots encourage both transparent reporting and meaningful inference. As supplementary material, we offer an 86-min YouTube tutorial^7^ together with the corresponding JASP files (including the data) that demonstrate how to create the plots presented below.

The following software was instrumental for implementing raincloud plots in JASP: R (R Core Team, 2023), ggplot2 (Wickham, 2016), ggrain (Judd et al., 2024), dplyr (Wickham et al., 2023), ggpp (Aphalo, 2024), and ggtext (Wilke & Wiernik, 2022). To ensure that all figures in this article are accessible to colorblind people, we used colorblindcheck (Nowosad, 2025).

### Example 1: Between-participant one-way ANOVA

For the first example, we simulated data in a between-participant one-way analysis of variance (ANOVA) design with three levels: Group A, Group B, and Group C. Each group features $\text {N}=50$ participants, each yielding a score on a dependent variable, for a total of $3 \times 50 = 150$ observations in the design.

**Fig. 3:**
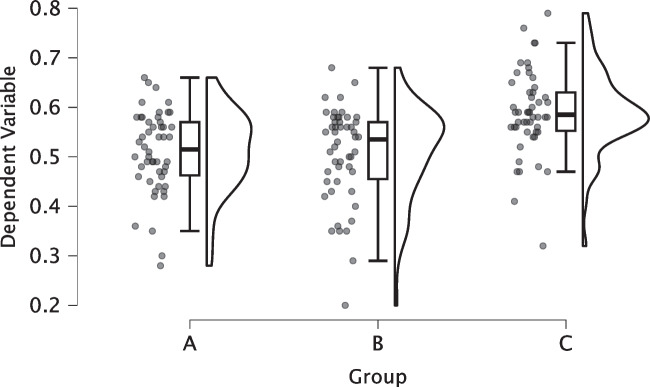
Standard descriptive raincloud plot of synthetic data from a between-participant one-way ANOVA design with three levels. Note: $\text {N} = 50$ per group. Figure from JASP (JASP Team, 2024)

A standard black and white raincloud plot of the synthetic data is shown in Fig. 3. Unlike the horizontal raincloud quartet (cf. Fig. 2), these rainclouds are now oriented vertically. Figure 3 provides an initial impression of the data: The dependent variable seems about equal in Groups A and B. In Group C, it seems a little higher. Furthermore, the plot reveals that the dependent variable is approximately normally distributed in each group. Finally, Group B and C each have a single observation that is notably low and might be classified as an outlier (for a Bayesian approach to outlier handling see Godmann et al. , 2024).

To test the differences between the three groups, we conducted a default Bayesian ANOVA (Rouder et al., 2012) in JASP (van den Bergh et al., 2020; JASP Team, 2024) where the prior Cauchy distribution on the fixed effects has a scale value of 0.5 (Wagenmakers et al., 2018). The resulting Bayes factor equals $\text {BF}_{10} = 5866.51$, which indicates extreme evidence in favor of a main effect (Lee & Wagenmakers, 2013; Jeffreys, 1961). Post hoc comparisons between the three groups revealed moderate evidence against a difference between Group A and B, $\text {BF}_{01} = 4.62$, but extreme evidence that Group C is higher both than Group A, $\text {BF}_{10} = 1726.36$, and than Group B, $\text {BF}_{10} = 1205.80$ (Lee & Wagenmakers, 2013; Jeffreys, 1961).^8^

**Fig. 4:**
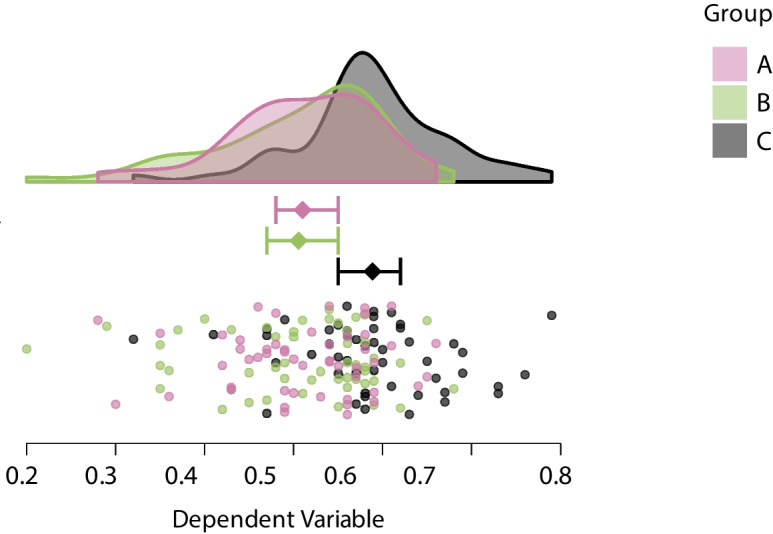
An overlapping raincloud plot of synthetic data from a between-participant one-way ANOVA design with three levels, emphasizing the difference in means across groups by including inferential content. *N* = 50 per group. Intervals around means show 95% credible intervals. Figure from JASP (JASP Team, 2024)

In order to add inferential content about this effect, the descriptive raincloud plot from Fig. 3 can be modified to produce Fig. 4. This figure plots the rainclouds horizontally, as overlapping densities with color coding. Moreover, it shows the sample mean of each condition instead of a box plot, as well as the 95% credible interval of each mean based on the statistical model that underlies the Bayesian ANOVA.^9^ Each interval can be manually specified, which allows the flexible visualization of inference from various statistical models and in combination with other software (e.g., an analysis of covariance in JASP).

Figure 4 emphasizes the difference between Groups A and B versus Group C in two ways. First, compared to Group A and B, the density estimate for Group C is shifted to the right. Second, with all three credible intervals next to each other, it is clear that the interval for the sample mean of Group C does not overlap with the other two. The data are now visualized in a transparent and informative way that encourages researchers to interpret this effect. For example, they may now discuss the size of the effect (Cumming & Calin-Jageman, 2024). In this specific scenario, the means (standard deviations) of the groups are $\text {M}_{\text {A}} =.51$ (.08), $\text {M}_{\text {B}} =.51$ (.10), and $\text {M}_{\text {C}} = .59$ (.09). Thus, the mean of Group C is about one standard deviation greater than that of Group A and B. According to Cohen (1992), this is a large effect.

**Fig. 5:**
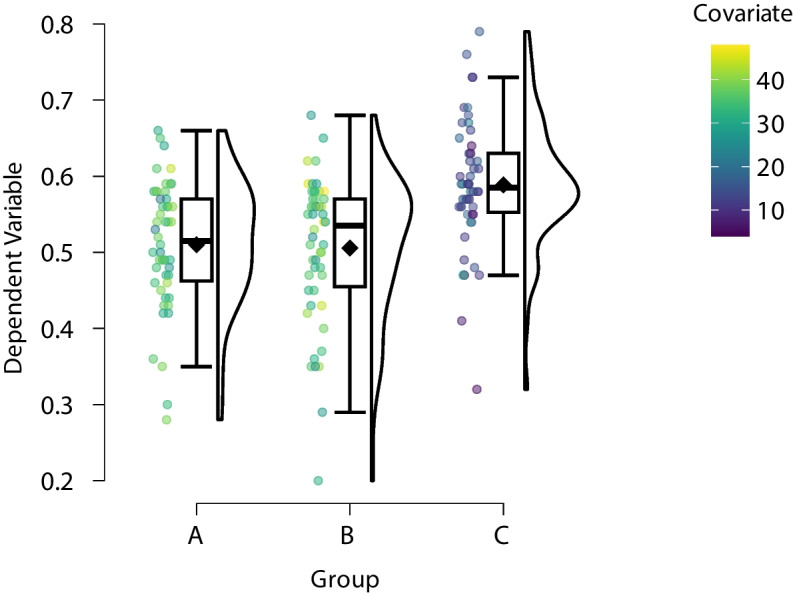
Descriptive raincloud plot of synthetic data from a between-participant one-way ANCOVA design with three levels and a confounding covariate. *N* = 50 per group. Figure from JASP (JASP Team, 2024)

The data set also comprises a continuous covariate that we have not considered so far. This continuous variable can be visualized in the raincloud plot by color coding. The resulting plot is shown in Fig. 5, which uses the standard raincloud representation but with the sample mean added. The plot provides two insights. First, observations beyond the box whiskers match their respective groups in regard to the covariate. In other words, the covariate does not offer a visually compelling explanation for why certain observations are suspiciously low or high. This might not be the case in other scenarios and should be explored. Second, the observations in Group C have lower values of the covariate compared to Groups A and B. This can be highly problematic as the difference between the groups may be caused by changes in the covariate (i.e., the covariate is a confounding variable). This illustrates how a raincloud plot can protect researchers against premature conclusions.

### Example 2: Mixed $2 \times 2$ ANOVA

For the second example, we use an adjusted subset of the synthetic data presented by Judd et al. (2024).^10^ These data concern the growth of sepal width for two species of flowers – versicolor and virginica – measured at two different points in time, before and after fertilizer treatment. This example was inspired by the iris data set (Anderson, 1935, as cited in Fisher, 1936). Specifically, in the hypothetical setup, an experimenter starts by measuring the sepal width of 50 versicolor flowers and 50 virginica flowers; these constitute the data before the treatment. Next, the experimenter applies a fertilizer; after some time has passed, the sepal width for each of the 100 flowers is then measured again; these measurements constitute the data after the treatment. In other words, each flower receives the fertilizer (the within-flower factor ‘fertilizer treatment’) but there are two separate species of flower (the between-flower factor ‘flower species’).

**Fig. 6:**
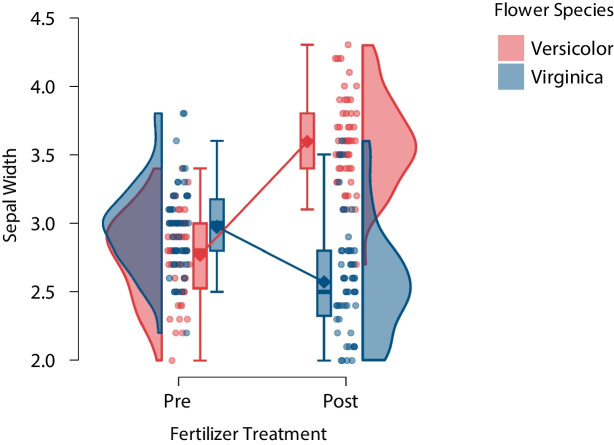
Raincloud plot of synthetic data suggesting that fertilizer increases the sepal width of versicolor flowers but decreases it for virginica flowers. $\text {N}_{versicolor} = 50$, $\text {N}_{virginica} = 50$. Figure from JASP (JASP Team, 2024)

The fertilizer data are displayed as a raincloud plot in Fig. 6. The plot emphasizes the mean change in sepal width for the two species and combines several features of the plots that were shown so far. The means and boxes are in the center of the plot and are connected via lines. The points and density estimates, on the other hand, are positioned on the outer sides so as to not overlap with the lines. This facilitates a visual comparison between the means: The plot suggests the presence of an interaction in the sense that the fertilizer *increases* the sepal width of versicolor flowers but *decreases* it for virginica flowers (we will not test this hypothesis statistically here). In addition, Fig. 6 shows that many observations share exactly the same value, suggesting that the data may have been obtained using a coarse, discrete-value measurement instrument.

**Fig. 7:**
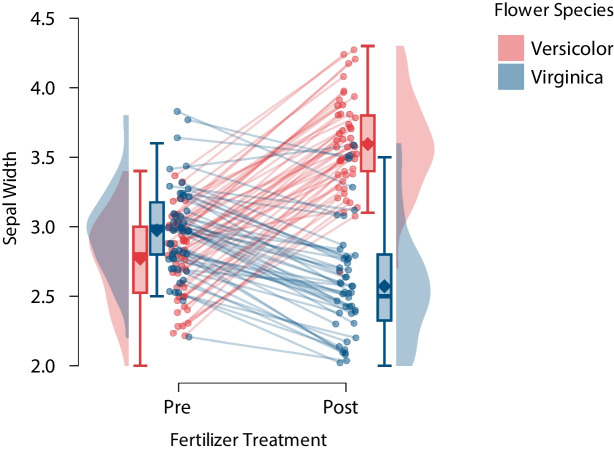
Raincloud plot of synthetic data emphasizing that the interaction effect for the means also holds for the vast majority of the individual flowers. $\text {N}_{versicolor} = 50$, $\text {N}_{virginica} = 50$. Points are slightly jittered from their true sepal width values to better discriminate individual flowers. Figure from JASP (JASP Team, 2024)

Finally, Fig. 7 presents a variation on Fig. 6, the main change being that lines connect the consecutive measurements for each flower. These individual trajectories highlight the fact that the interactive pattern for the group means also holds for the majority of the individual flowers. The individual data points are slightly jittered from their true sepal width values in order to reduce the visual overlap and allow for an easier discrimination. The means and boxes are now positioned further out so as not to overlap with the lines in the center. Also, to draw attention to the individual observations in the center, the opacity of the densities and boxes is decreased and the outlines of the densities are removed. Because Fig. 7 focuses on the change for individual units, this general type of plot may be particularly informative for hierarchical models that feature random effects in participants and items.

## Conclusion

Raincloud plots (Allen et al., 2021) allow researchers to visualize and report their data in a comprehensive fashion that goes substantially beyond the standard bar plots and line plots. A focused literature review of research in cognitive experimental psychology revealed a strong trend towards transparent, informative data visualization. In 2023, 9% of plots in Brief Reports from *Psychonomic Bulletin & Review* were raincloud plots. Another 29% of plots (vs. 2% in 2013) present an intermediate step in this development as they show raw data, that is, the individual observations.

In order to make the advantages of raincloud plots available to a broad audience of empirical scientists and their students, we implemented a comprehensive suite of raincloud plotting options in JASP (JASP Team, 2024). Examples from two popular factorial designs underscored the added value of raincloud plots for exploring, analyzing, and reporting empirical data.

A future avenue of raincloud plotting could be their comprehensive adaptation to dependent variables that are discrete rather than continuous (e.g., the Likert-data plots in Hoogeveen et al. (2023)). It should be kept in mind that raincloud plots might not always be the best choice: if a design has factors with many levels (e.g., a $6 \times 4$ design), then the different raincloud elements might clutter the plot and obscure the central findings that researchers wish to communicate. Nevertheless, even in such cases, raincloud plots could still be useful for initial data exploration.

When researchers decide to adopt a specific data visualization tool, we believe that their choice should be dictated by the science rather than the software. With the JASP GUI, it is now easy to create raincloud plots, and this means researchers can make a conscious choice whether or not to report them. Until recently, students and researchers who lacked either time or programming expertise could get raincloud plots “only with trouble, cunning and a fighting spirit” (Anscombe, 1973). With the recent implementation of raincloud plots in JASP, this has finally changed.

## Data Availability

All data, code, and JASP files can be found at https://osf.io/mh2ka/?view_only=b87b620f056f4143b550c62e154195d4. A 86-min YouTube tutorial on how to create raincloud plots in JASP can be found at https://www.youtube.com/watch?v=AAdXUAl_w6E.

## Footnotes

1 This raincloud quartet was created via simulated annealing (Matejka & Fitzmaurice, 2017) with the help of the R packages moments (Komsta & Novomestky, 2022) and mousetrap (Wulff et al., 2023), the work by Blanca et al. (2013) and Freeman and Dale (2013), and ChatGPT (Brown et al., 2020).

2 No other volumes or journals were assessed. We focused on Brief Reports because these almost always present new empirical data, whereas this is not the case for the longer Theory & Review articles.

3 https://huygens.science.uva.nl/SuperPlotsOfData/

4 https://lcdlab.shinyapps.io/raincloudplots-shiny/

5 https://shiny.hiplot.cn/raincloud-shiny/

6 See for instance the JASP world map at https://jasp-stats.org/world-map/.

7 https://www.youtube.com/watch?v=AAdXUAl_w6E

8 Corrected for multiple testing (Westfall et al., 1997), the prior probability of .37 for a difference between any two groups thus changes. It becomes less probable that groups A and B differ (posterior probability = .12), but highly probable that Group C is higher than Group A and B (both posterior probabilities near 1).

9 If desired, frequentist confidence intervals can also be displayed.

10 See the vignette at https://cran.r-project.org/web/packages/ggrain/vignettes/ggrain.html.
